# Exploring Ethnic Disparities in Burn Injury Outcomes in the UK: A Systematic Review

**DOI:** 10.3390/ebj6030048

**Published:** 2025-08-22

**Authors:** Uashar Badakhshan, Reza Zamani, Tanimola Martins

**Affiliations:** Medical School, Faculty of Health and Life Sciences, University of Exeter, Exeter EX1 2LU, UK; ub218@exeter.ac.uk (U.B.); tom207@exeter.ac.uk (T.M.)

**Keywords:** burns, ethnicity, scalds, first aid, outcomes, incidence

## Abstract

Background: Burn injuries are among the most distressing injuries, affecting approximately 250,000 people annually in the UK. While extensive research has explored how gender, health, and socioeconomic factors influence burn injury rates, ethnic disparities in burns have received less attention. Methods: The review followed the PRISMA framework for database searches. Search terms included concepts of ethnicity and burn injuries in the UK. Results: From the initial 3339 search results, 11 studies were selected following the eligibility screening. White ethnic groups made up 52.4% of admissions, whereas Asians and Black groups made up 24.9% and 5.9%, respectively. Trends showed a decline in admissions among White patients and a rise in admissions among Black patients. Children aged 1 to 5 years were most affected, particularly in the Asian and Black groups. Males constituted 58.0% of admissions, while females accounted for 42.0%. Most burns occurred at home, with scalds, particularly among children. Ethnic minorities were more likely to apply unsuitable topical treatments and had higher rates of psychological referrals. Conclusions: The review underscores important ethnic disparities in burn injuries and outcomes. Targeting policies to address them could result in a more equitable healthcare system and improved outcomes for burn patients in the UK.

## 1. Introduction

Burns are among the most physically and psychologically devastating injuries a person can experience. They occur when the skin and soft tissue are damaged by exposure to heat, cold, electricity, chemical contact, friction or radiation (electromagnetic or ionising) and may require specialist care for their treatment [1,2]. In the UK, an estimated 250,000 people sustain burn injuries each year, with hospitalisation rates of approximately 18 per 100,000 people annually in England and Wales [3,4]. For more severe burn injuries requiring hospital stays of 72 hours or more, the rate is 4.7 per 100,000 people per year. The severity of burns is often classified by the depth of tissue damage and the extent of the burn, according to the Wallace Rule of Nines or the Lund and Browder chart, measured as a percentage of the total body surface area (TBSA) [5]. A typical hospital admission for a minor burn (involving less than 10% of the TBSA) costs the National Health Service (NHS) approximately £1850 per case in 2010, whereas major burns (involving 30–40% of the TBSA) can cost up to £63,157 in children [6]. The long-term effects of burns are more evident in children, in some cases leading to psychosocial problems and a loss of bone density in severe cases [7]. There is extensive research into how gender, health and socioeconomic status are associated with rates and extent of burn injuries, but less focus has been given to ethnic differences in burn experience [8]. Therefore, there is a growing interest in understanding how sociodemographic determinants, such as ethnicity (also referred to as ‘racial group’), may be related to burn outcomes.

Despite ongoing efforts to improve public health, disparities continue to persist across the UK. A recent study found that certain ethnic minority groups, including White Gypsy or Irish Travellers, Bangladeshi and Pakistani communities have the poorest health outcomes in England [9]. Alongside this, ethnic minorities are at significantly greater risk of sustaining burn injuries in the UK, although the basis of this variation is not fully understood [10]. This trend underscores the importance of culturally appropriate prevention and treatment, as well as targeted education and training of healthcare providers. However, international clinical guidelines such as those from the World Health Organisation or the UK’s National Institute for Health and Care Excellence do not explicitly recognise ethnicity as a risk factor for burn injuries [3,11]. This omission is notable given the significant ethnic disparities observed in burn outcomes. The underlying causes of these disparities, particularly among Black populations and other ethnic minority groups, remain poorly understood and are likely shaped by a complex interplay of genetic, cultural, and socioeconomic factors [12]. For example, certain individuals may be more susceptible to burns due to occupational exposures or household practices involving hot liquids. What is less clear is why certain ethnic groups have a higher prevalence of such risks [13]. In addition, barriers in language and culture may impact communication and trust between patients and healthcare providers, affecting treatment and health outcomes [14].

Currently, there is a lack of comprehensive synthesis of evidence regarding ethnic disparities in burn presentation and outcomes in the UK. While countries such as the United States have documented disparities in burn treatment linked to ethnicity, similar findings have yet to be reported in the UK [15]. The present study aimed to address this gap by reviewing available evidence on the epidemiology, presentation, and outcomes of burns among ethnic communities in the UK, with a focus on identifying potential barriers to accessing healthcare services.

## 2. Materials and Methods

The methodology of this systematic review follows the Preferred Reporting Items for Systematic Reviews and Meta-Analysis (PRISMA-DTA 2020) guidelines [16].

### 2.1. Literature Searches

The search terms relating to ethnicity, burns and the UK were combined with the following Boolean operators: Ethnicity: “Ethnic” or “Race” or “Black” or “Asian” or “Minority” or “Mixed” or “Other” or “BAME” AND Burns: “Burn” or “Burn patient” or “Burn outcome” or “Burn access” or “Burn barriers” or “Burn treatment” or “Burn management” AND the UK: “United Kingdom” or “Britain” or “England” or “Wales” or “Scotland” or “Northern Ireland”. Further variations in search terms related to ethnicity were used according to the NHS ethnic category code [17]. All the searches were performed in PubMed, TRIP, Web of Science, Scopus, Ovid (Medline/Embase) and EBSCO databases to find relevant articles on 22 October 2024, with the process repeated on 2 August 2025.

### 2.2. Study Selection Criteria

Primary research that recorded or investigated the association between burns and ethnicity in the UK was reviewed. No limit was set on the date of publication. All included studies focused on burns-related admissions or outcomes within NHS secondary care settings, and most employed observational or cross-sectional designs. The Population, Exposure, Comparator and Outcomes (PECO) criteria were used to formulate the inclusion and exclusion criteria for the articles [18]. The study population comprised individuals in the UK who had sustained burn injuries, with documented information on ethnicity, age, and gender. The exposure group comprises individuals from an ethnic minority background, encompassing groups such as Asian, Black, Mixed, and other non-White ethnicities. This included both UK-born and migrant individuals. Comparisons were made across ethnic groups, including the White British population, and further stratified by age and gender where relevant. Only studies that reported one or more of the following primary outcomes were included: mechanism and severity of burns (e.g., scalds, flame burns, total body surface area affected), mortality, complication rates, duration of hospitalisation and recovery, appropriateness of first aid measures, access to psychological support services, and long-term physical and psychosocial outcomes. Studies were excluded if they did not clearly define or stratify outcomes by ethnicity, or if ethnicity was not a primary or secondary variable of interest.

### 2.3. Screening

The screening process was conducted using Rayyan (2024 version), with the automatic deduplication function. This process was also manually cross-checked for accuracy. Following this, the titles and abstracts of the articles were first screened by two authors (U.B. & R.Z.) to remove any ineligible studies (e.g., conference abstracts). For any studies where consensus could not be reached, the full text was independently reviewed by the third author (T.M.) to determine eligibility (see ‘Author’s Contributions’ for more information).

### 2.4. Data Extraction & Analysis

A narrative synthesis approach was employed to analyse findings across the included studies. Due to the heterogeneity of data collection, populations, and outcomes, a meta-analysis was not feasible. Instead, findings were grouped thematically and summarised across key domains including cause, type and extent of burns, demographic patterns (age, gender, ethnicity), and geographic or environmental factors. Where studies reported similar designs, population groups, and outcome measures, the results were combined as appropriate. The results were further organised into subgroups based on participants’ age, gender and ethnicity, allowing for exploration of disparities in burn incidence and outcomes. Timeline trends were also examined using data extracted across study timelines to contextualise shifts in incidence by ethnicity and gender over time. Alongside this, statistical significance from individual studies (e.g., *p*-values) was reported to highlight key associations and differences. For the data recorded and statistical tests used, a *p*-value < 0.05 was considered statistically significant. The NHS ethnic category codes were used to classify individuals wherever possible; when this was not feasible, broader ethnic group classifications as reported in the original studies were used.

### 2.5. Quality Appraisal

The quality of selected studies and the risk of bias were assessed by adopting the Critical Appraisal Skills Programme (CASP) checklist for cohort and qualitative studies. Each checklist consists of ten questions that evaluate the internal and external validity of each study [19]. Each question offers three response options: “Yes,” “Can’t tell,” and “No,” depending on whether the study meets the CASP criteria. A scoring method was applied, assigning ‘Yes = 2’, ‘Can’t tell = 1’ and ‘No = 0’, resulting in an overall score ranging from 0 to 20, with higher scores indicating higher quality. Additionally, journal ranking, including the quartile, sources of research funding and statements about conflict of interest were taken into consideration. The studies were then divided into three quality levels: low quality (0–13), medium quality (14–17) and high quality (18–20), as indicated in Table S1 (see Supplementary Data).

## 3. Results

### 3.1. Identification of Studies

**Figure 1:**
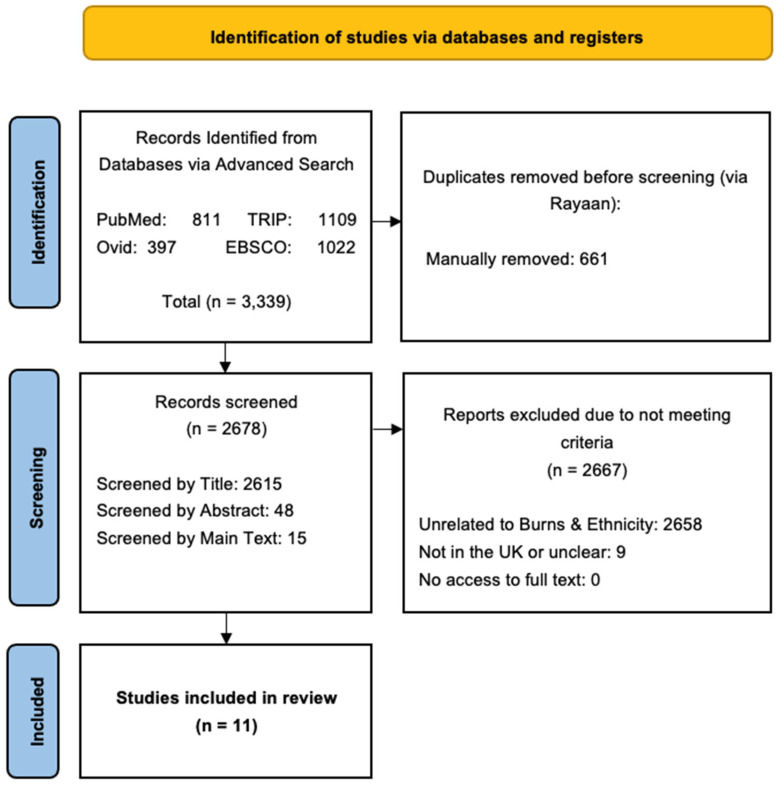
PRISMA flow diagram. Chart shows inclusion screening process for articles identified by our searches (**left panels**) and a breakdown of exclusion reasons (**right panel**).

The initial search using databases PubMed, TRIP, Ovid (Medline/Embase) and EBSCO yielded 3339 articles from the four databases. No additional results were retrieved from the second search on 2 August 2025 (see Section 2.1), or from either Web of Science or Scopus during the search process, and after removing duplicates, 2678 articles remained. Of these, 2667 were excluded for not meeting the eligibility criteria, leaving 11 articles for final analysis. A comprehensive list of the search terms used in PubMed, TRIP, Ovid and EBSCO is provided in Table S2 (see Supplementary Data). A PRISMA flowchart showing the screening process and reasons for study exclusion is illustrated in Figure 1.

### 3.2. Study Characteristics

**Figure 2:**
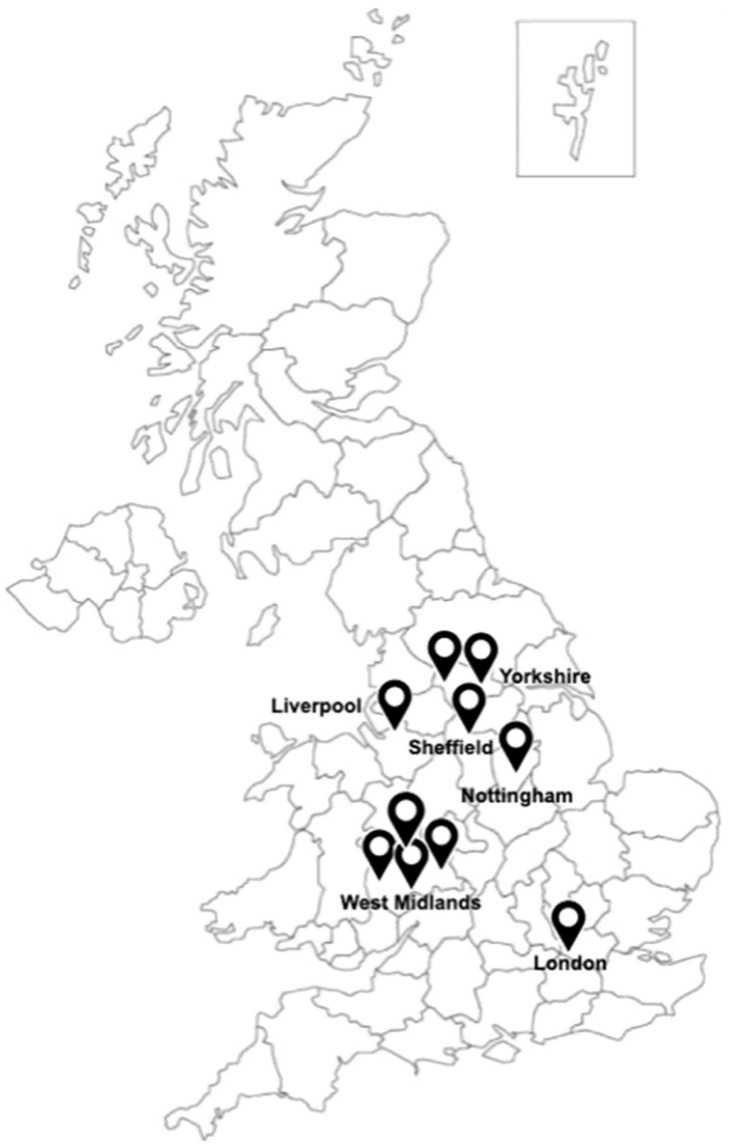
Approximate locations of studies included in the review [31].

The characteristics of the included studies are summarised in Table 1. Ten studies used data from primary research [20,21,22,23,24,25,26,27,28,29], while one study involved a secondary analysis of data from previous work [30]. Of the eleven identified studies, six [20,21,22,23,24,25] specifically focused on burns in the infants, children and young adult population (i.e., under the age of 18), while the remaining five included participants of all age groups. The ages ranged from infants less than one year old to 97 years. Four studies were conducted within the last 10 years [21,23,27,29], seven in the last 15 years [20,21,22,23,24,26,27,29] and one study was conducted in 1989 [25]. In total, the 11 studies included 196,951 individuals. Nine of the studies reported gender, accounting for 4721 males (57.8%) and 3445 females (42.2%) [20,21,23,24,25,27,28,29,30]. The remaining two studies involving 188,798 patients [22,26] did not report gender. Of the 9 studies (including 5810 participants) where ethnicity was recorded, the majority were White British or Other White (55.8%), followed by Asian or Asian British (21.8%), Black British or Black Caribbean or Afro-Caribbean (5.5%), Mixed (1.0%), and Other (1.3%). Individuals recorded as ‘Unknown’ comprised 14.6% of the review sample. All 11 studies used ‘White’, ‘White British’, ‘Caucasian’ or ‘Non-Asian’ as the reference ethnic group for comparison and were conducted in England (Figure 2).

#### 3.2.1. Cause of Burns

Scalds were the leading cause of burns across all ethnic groups (n = 4923), accounting for 64.1% of cases in seven of the studies [20,21,23,24,25,28,30], followed by contact (18.6%), flame (7.5%), other (3.4%), flash (2.3%), chemical (1.5%), electrical (0.6%), friction (0.3%) and ultraviolet radiation (0.2%). The remaining were unknown (1.6%). Children who sustained flame and flash burns were significantly older (mean age = 8 years and 11 years, respectively) than those sustaining scald or contact burns (mean age = 3 years and 1 month, respectively) [23]. In one study, White children accounted for 75% of all the bathing-related cases [20]. Among Chinese children, 60% of burns were due to hot food, 27% from hot beverages, and 13% from hot water [24].

#### 3.2.2. Type & Extent of Burns

Burn injuries affecting less than 10% of the TBSA were reported in the majority of the five studies (n = 3969) [20,21,27,28,30]. Children sustaining flame burns had a higher mean percentage TBSA (13.4%) compared to those with flash burns (5.7%) and scalds (6.0%) across all ethnicities [24]. The hand and wrist were the most frequently affected regions of the body among Asian patients (38%), compared to non-Asian patients (34%) [28]. Ethnicity-based analyses yielded conflicting findings. For instance, in a study by Alnababtah et al. examining children in the West Midlands, there was no significant difference in mean percentage TBSA between ethnicities: White (5.7%, n = 706), Asian (4.7%, n = 309), Black (4.8%, n = 62), and Mixed (4.2%, n = 28) [20]. In contrast, a study by Tan et al. in Liverpool found significant differences in mean percentage TBSA among children of different ethnicities: White (5.6%, n = 692), Asian (8.2%, n = 20), and Chinese (10.2%, n = 16) [24].

#### 3.2.3. Proportion of Burns

**Figure 3:**
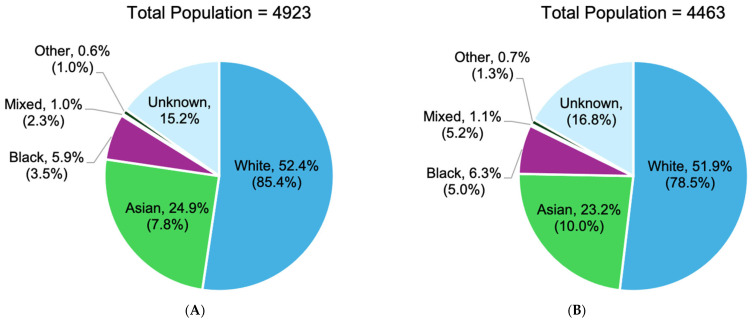
Proportion of burns by ethnicity (in percentage) of White (Range: 78–787), Asian (Range: 35–456), Black (Range: 9–169), Mixed (Range: 5–34), Other (12–18) and Unknown (303–445) ethnic groups in burn cases within England [20,21,23,24,25,28,30]. () = the percentage of the ethnic group population residing within England based on data from the 2011 Office for National Statistics (ONS) census [32]. (**A**) = represents data from all age groups from 1987 to 2012 (n = 4923); (**B**) = represents data from children only from 2004 to 2012 (n = 4463).

**Figure 4:**
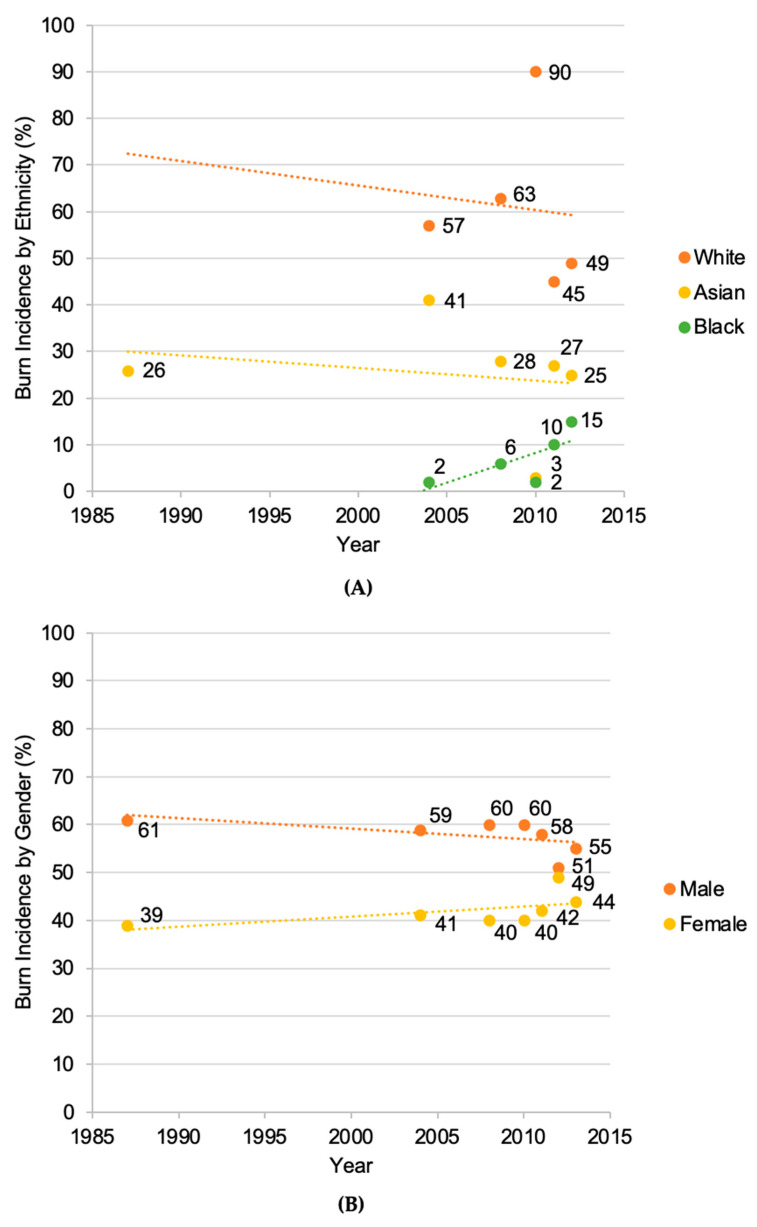
Chronological order of burn incidence by the three most common ethnicities from 1987 to 2012 (n = 4129) and gender (n = 7008) from 1987 to 2013 in England [20,21,23,24,25,27,28,30]. The linear dotted line, generated as a linear trendline in MS Excel, illustrates the trend in burn admissions as a percentage for each category. (**A**) = White, Asian and Black; (**B**) = Male and Female.

The proportion of burn injuries varied significantly across ethnic groups in England over the study period [20,21,23,24,25,26,27,28,30]. Specifically, analysis of seven studies revealed that White patients accounted for 52.4% of total admissions, followed by Asian patients (24.9%), Black patients (5.9%), Mixed ethnicity (1.0%), Other ethnic groups (0.7%), and those with unknown ethnic group status (15.2%) (Figure 3). Figure 4A shows an apparent decline in the proportion of burn injuries within the White population. In contrast, the proportion among the Asian population remained relatively stable, while cases in the Black population showed a general increase. Analysis by gender revealed that males accounted for 58.0% of cases, whereas females represented 42.0% of cases across 9 of the 11 studies [20,21,23,24,25,27,28,30]. Figure 4B reveals a decline in male burn injuries over more recent years, while the proportion among females indicates a steady increase over the same period.

Children constituted up to 75% of admissions in both the Asian and Black groups (age range from 3 days to 16 years) in five studies [20,21,23,24,25]. Asian children with burn injuries were significantly younger on average than White cases (*p* < 0.0001), with mean ages of 3 years and 1 month (n = 352) and 4 years and 5 months (n = 787), respectively [20]. The age-standardised rates of admissions for burns across England were higher in the non-white minority groups compared to the white group [26]. The incidence of childhood burns was found to be significantly higher (*p* = 0.001) among children from ethnic minority families with single parents compared to those with single White parents in one study [21].

#### 3.2.4. Location of Burn Incidents

Four studies reporting the location of burn incidents found that over half occurred within the home environment (n = 1386) [21,24,28,30]. In one study, Asian individuals experienced a higher proportion of indoor burns (91%) compared to White individuals (82%) [28]. The kitchen emerged as the most common site for burn injuries, with rates ranging from 36% to 56% [24,28,30]. Children from ethnic minority groups were more commonly affected by burn injuries in the kitchen: 100% of Arab children’s burns occurred in the kitchen, compared to 63% in Black children, 54% in Chinese children, and 34% in White children [24]. Other frequently reported locations for burn injuries include the living room (22–24%), the bathroom (4–9%), and the bedroom (7–13%) [24,28,30].

#### 3.2.5. First Aid Before Admission

Three of the studies reported that the proportion of patients who received first aid for burns ranged from 29% to 60% [23,28,30]. First aid provision for burns was low across all ethnic groups, with some patients receiving no treatment before presentation to Accident and Emergency (A&E) [23,28,30]. The rates of first aid did not differ substantially by ethnicity, ranging from 60.2% in Asian patients (n = 456) and 62.5% in Black patients (n = 169) to 69% in White patients (n = 760) [23]. 39% of White parents reported that they would cool a burn under running water for the recommended duration, compared to 19% of parents from other ethnicities (*p* = 0.05) [22]. Ethnic minorities were significantly more likely to use inappropriate topical agents, such as toothpaste, butter, milk, or cooking oil, in 11% of cases (n = 36, *p* < 0.05) [22,28]. Additionally, one study reported that pre-hospital analgesia was infrequent, with only 10% of Asian patients and 13% of non-Asian patients receiving pain relief prior to admission [28].

#### 3.2.6. Length of Stay in Hospital

Two studies examined the length of hospital stay following burn injuries, with Tan et al. demonstrating that ethnic minority children had significantly longer hospital stays compared to White children (*p* < 0.001) [24]. Among survivors, hospital stays ranged from 0 to 162 days, with a mean of 4.2 days in the same study. In contrast, Alnababtah et al. found no significant differences in the duration of hospital stay between White (5.1 days ± 26.3), Asian (3.8 days ± 21.9) and Black patients (2.9 days ± 4.2) [20]. The mean percentage TBSA was comparable across all ethnicities: White 5.7 (± 9.0), Asian 4.7 (± 7.3), and Black 4.8 (± 5.6) [20]. Moreover, hospital stays were significantly longer for flame and flash burns compared to scalds, with mean durations of 14.4 days for flame burns, 4.9 days for flash burns, and 3.8 days for scalds (*p* < 0.001) [24].

#### 3.2.7. Outcomes Following Admission

One study reported on the clinical management of patients following a burn, while another examined differences in psychological assessment after burn injuries. 54% of patients received follow-up care in the A&E return clinic, while 3% of Asian patients and 1% of non-Asian patients were referred to plastic surgery services [28]. One Asian patient and four non-Asian patients required skin grafts for their burns and three Asian patients and nine non-Asian patients were transferred to the Regional Burn Centre for further treatment. No patient deaths were reported in this cohort [28].

White patients were less likely to be referred to psychological services, with 471 referrals out of 1135 burn-related admissions. In contrast, referrals were significantly higher among ethnic minority groups, with 28 referrals from 33 Black admissions (4%, *p* < 0.001) and 43 referrals from 57 Asian admissions (6.2%, *p* < 0.001) [29]. There were higher-than-average rates of patients declining a psychological assessment following a burn among ethnic minority groups. Specifically, 14 Asian patients (33%), 9 Black patients (32%) and 5 Mixed patients (50%) declined psychological assessment, compared to 125 White patients (25%). However, this difference was not statistically significant (*p* > 0.05) [29].

## 4. Discussion

### 4.1. Culturally Appropriate Prevention

The most effective way to address burns is through the reduction of risk factors that lead to such incidents; this requires a culturally sensitive approach, including traditional practices, health education, recognising burns on different skin tones, and ease of access to healthcare services. Therefore, this discussion focuses on strategies that can be integrated into public education and policies. This review aimed to identify individuals and communities at the highest risk of burn injuries. It is well established that young children, elderly people and those with health-related disabilities such as dementia, learning difficulties, and reduced mobility are more susceptible to burns than the general population [33]. However, the current findings reveal that individuals from ethnic minority groups, particularly those of Asian and Black descent, showed higher associations with burn injuries than their White counterparts, with Black patients demonstrating a consistent rise in burn admissions over time. However, these findings likely vary across different ages and geographical regions. For example, White children accounted for 52% of burn cases; this is disproportionately lower than their age-matched population, while Black and Asian children appeared overrepresented relative to their respective population proportions. Similarly, Richards et al. found that Asian children represented 27% of burn cases in the West Midlands, despite constituting only 17% of the 0 to 15-year-old population in the region, while Black children accounted for 10% of cases, despite making up just 4% of the total population [23]. This issue was first highlighted by Vipulendran in 1989, who observed a disproportionate number of Asian children admitted to burn units in Birmingham with scald injuries [25]. On a global scale, Asian and African populations have been shown to experience higher-than-average rates of burn injuries [34].

Burns frequently occurred in the kitchen, with a particularly higher incidence among the Asian population and children. These findings suggest a possible link with cultural cooking practices, which often involve the use of hot liquids, the most common cause of burns found in this review. This pattern is consistent with reports from national studies, which highlight scalds as a predominant cause of burning among all ethnic groups, including the White population [35]. Thus, given the rising incidence of burns across various ethnic groups and the increasing multicultural demographic in the UK, there is a pressing need for tailored prevention programmes. Such programmes should aim to offer culturally appropriate safety alternatives and educate the public on effective burn prevention measures within the domestic and occupational settings.

Local authorities play a crucial role in burn prevention. Many local fire services offer free safety checks aimed at preventing and educating residents about fires. However, these events are often targeted towards elderly people or those with long-term illnesses [36]. A potential strategy could be to extend these services to residents in areas with higher levels of deprivation and diversity. In October 2023, new fire safety regulations were introduced, mandating that buildings be designed and constructed to limit the spread of fire and facilitate safe evacuation [37]. These measures aim to prevent tragedies such as the Grenfell Tower fire, where many of the victims were of Middle Eastern, Black or Asian heritage [38]. Additionally, research indicates that certain ethnic groups are disproportionately represented in occupations with a higher risk of burn injuries, such as construction or food services [39,40]. As such, individuals in these roles should receive comprehensive safety training, and employers are legally required to conduct fire risk assessments [41,42]. Furthermore, a recent study on the rise of acid attacks revealed that Western Europeans (36%) were the most affected by chemical assaults, followed by individuals of African/Caribbean descent (24%) and Asian descent (23%) [43]. These findings highlight the need for collaboration with community leaders, health educators, and policymakers to create inclusive and effective burn prevention strategies.

### 4.2. Improving Equity and Outcomes

Burn injuries represent a significant public health challenge, with outcomes influenced by the quality and accessibility of care [44]. In the UK, the healthcare system is predominantly provided by the NHS and offers specialised burn care through regional burn centres equipped with multidisciplinary teams [45]. Despite this, disparities in the treatment and outcomes among different ethnic groups have become increasingly evident [46]. These inequalities may stem from a range of healthcare-related factors, including ideology, cultural beliefs, and implicit biases related to gender, age, ethnicity, socioeconomic status, or body weight [47].

The data regarding differences in burn severity among ethnic groups remains inconclusive. While Tan et al. reported a significant difference in TBSA across ethnic groups of children (*p* < 0.001), Alnababtah et al. found no such variation (*p* = 0.2) [20,24]. However, the generalisability of these findings is limited. In Tan et al.’s study, fewer than 10% of participants were from non-White ethnic backgrounds (n = 58) [24]. Alnababtah et al., on the other hand, only included children who were referred to and admitted at that burns unit, excluding those treated in emergency departments, minor injury units, or managed in the community, which may conceal any differences in these groups [20]. Given these methodological limitations, the reported differences in TBSA may not accurately reflect patterns in the broader population. Moreover, ethnic minority children were reported by Tan et al. to have significantly longer hospital stays than White children did (*p* < 0.001) [24]. Conversely, Alnababtah et al. reported no significant difference in the duration of hospital stay among White, Asian, and Black patients [20]. These conflicting findings, coupled with a lack of comprehensive data, make it difficult to draw firm conclusions about ethnic disparities in hospital stay length.

Data on tertiary care for extensive burns and their long-term outcomes are limited, with only two studies identified in this review. Rawlins et al. reported that only three Asian patients and nine non-Asian patients were transferred to the Regional Burn Centre [28]. Regional variations in NHS budgeting and healthcare delivery may also contribute to differences in outcomes [48], complicating the assessment of disparities in referrals to specialised burn centres.

While the psychological ramifications of burns may be profound, evidence in this area remains limited. Black, Asian, and Mixed ethnic patients have been shown to have higher rates of declining a psychological assessment following burn injuries [29]. This may stem from a broader range of issues, as patients from ethnic minority backgrounds often face significant challenges in accessing mental health services, and may reflect cultural differences, stigma, or mistrust in healthcare professionals, highlighting a critical area for future research [49]. In the United States, multiple studies have examined burn care issues by ethnicity, highlighting differences in pain perception, complication rates, hospital stays and acceptance of skin grafts among certain groups [15]. However, research on pain and treatment differences by ethnicity in the UK remains limited, making it unclear whether similar differences exist. The underrepresentation of ethnic minorities in burn-related research can contribute to a lack of tailored treatment protocols that account for genetic, physiological and cultural underpinnings [50,51]. Addressing these research gaps could promote a more equitable healthcare system and help mitigate the effects of systemic barriers on burn injury outcomes.

The National Burn Injury Database (NBID), which includes data from Hospital Episode Statistics (HES) and its operational arm, the International Burn Injury Database (iBID), provides epidemiological data on burn injuries in the UK. However, these databases are not publicly accessible, and are unable to determine their scope or data quality, nor include them in the present review. Nonetheless, Brewster et al., using HES data, found that hospital admission rates for burn injuries in England were higher among most ethnic minority groups compared to the White population between 2001 and 2010 [26]. Thus, this highlights not only the ethnic disparities in burn injury burden but also the need for more transparent and accessible data to enable robust research.

### 4.3. Future Challenges

Addressing ethnic disparities in burn injuries poses several challenges. One key is the anticipated increase in demographic diversity, which will require the healthcare system to adapt to a broader range of cultural and linguistic needs [14]. Notably, over 14% of burn cases in this study were recorded as having ‘unknown’ ethnicity, highlighting a significant gap in NHS data collection, and limiting a fuller assessment of disparities in burn injury presentation, and outcomes [52]. Although NHS England provides guidance on recording patients’ ethnicity within clinical settings [53], inconsistent implementation remains a challenge. Furthermore, multiple studies categorised patients’ ethnicity with insufficient granularity, such as using broad terms like “Asian” without specifying the ethnic sub-groups included, which restricts the ability to determine which specific ethnic communities may be more vulnerable to burn injuries. Consequently, conclusions drawn from such aggregated data may overlook meaningful disparities and reduce the overall interpretability of this review. This inconsistency in data presents a unique challenge for developing tailored healthcare planning for the ethnic groups, who may be more susceptible to burn injuries with or without significant TBSA. Future research should adopt standardised approaches to the classification of ethnicity, such as the NHS ethnic category code [17] to enable more accurate and equitable evaluations of outcomes across diverse populations. As a result, in this paper, for comparison purposes, ethnicity was classified using the Office for National Statistics (ONS) data categories: White, Asian, Black, Other or Mixed. Most of the studies were conducted in UK regions with higher-than-average levels of diversity, notably the West Midlands and the North of England (Figure 2). However, our final selection included only studies conducted in England, excluding those from the devolved nations—Scotland, Wales, and Northern Ireland. While this geographic limitation may affect the generalisability of our findings to the UK as a whole, we do not anticipate substantial differences in the patterns or outcomes related to burn injuries across these countries, given the similarities in healthcare systems, population demographics, and service delivery structures.

Several factors remained independently associated with the relative risk of paediatric domestic burns, including the percentage of ethnic minorities, income deprivation (*p* < 0.001), health deprivation and disability scores, the proportion of families with three or more children, and barriers to housing and services (*p* < 0.05). Tan et al. highlighted that ethnic minority children were significantly more deprived than their non-ethnic minority counterparts (IMD 48.7 vs. 40.9; *p* = 0.02) [24]. Similarly, Alnababtah demonstrated that the distribution of deprivation varied across ethnic groups (*p* < 0.002), and that burn injuries in children were significantly associated with families living in social accommodation (*p* < 0.0005) [21]. While these findings are important, they lack the granularity necessary for effective health policymaking. This underscores the importance of future research capturing ethnicity alongside other key social determinants of health, such as socioeconomic status, education level, housing quality, healthcare access, and occupational risk to better inform prevention strategies and policy development. These factors should be further stratified to address the current lack of comprehensive data on psychological and long-term outcomes from burns in the UK.

While advancements in burn assessment and treatment are promising, they may not be equitably accessible to all ethnic groups, particularly those from lower socioeconomic backgrounds [54,55]. Therefore, greater emphasis is needed on studies examining variations in burn treatment based on ethnicity to determine whether disparities exist. The provision of first aid before hospital admission is often inadequate across all ethnic groups. Notably, Asian patients are less likely to receive effective first aid, such as cooling the burn under running water or removing hot clothing. Furthermore, ethnic minorities are significantly more likely to use inappropriate topical agents, such as toothpaste, butter, milk, or cooking oil, potentially exacerbating the injury. Encouragingly, in 2020, the Department for Education mandated that all state-funded schools teach first aid as part of health education [56]. As a result, primary school pupils must be taught basic first aid skills, such as how to recognise and treat burns. However, concerns remain about whether the depth and quality of this education are sufficient to ensure safe and effective burn first aid practices [57].

### 4.4. Risk of Bias

This study examined multiple factors and considered diverse perspectives in burn injury research in the UK. However, not all major regions were included due to a lack of published research including ethnicity in burn research. The inclusion of various demographic factors, such as gender, age and ethnicity, provides a comprehensive understanding of burn epidemiology. As a result, future interventions may target certain communities to reduce the incidence of burns and/or provide training in first aid treatment at home. However, there are limitations to our report, including the potential for incomplete or biased data due to the underreporting of ethnicity information in NHS records. Additionally, despite accessing a range of databases, there is a possibility that some relevant published research may not have been captured. The lack of studies recording ethnicity accurately may have also led to inconsistencies in interpreting the results and the applicability of the study’s conclusions. Moreover, the study also does not fully account for confounders like socioeconomic and educational attainment factors that may contribute to burn injuries or the long-term outcomes of patients with burns. The quality appraisal rated three studies as high quality [23,27,29], five as medium [20,21,24,26,30], and the remaining studies were classified as low quality [22,25,28]. No conflicts of interest were reported by any research reports selected in this study, though this information was absent in three of the studies (see Table S2, Supplementary Data).

Many of these studies were among the first to explore and document the relationship between ethnicity and burns, making it challenging to compare their findings with those from other regions. Additionally, most studies did not adequately follow up with patients after a burn injury, making it difficult to evaluate differences in treatment quality by regions and outcomes, based on ethnicity. In addition, multiple studies failed to follow the NHS ethnic category codes, making it difficult to compare outcomes amongst certain ethnic groups [25,26,27]. Moreover, the data was not analysed from the perspective of healthcare practitioners’ training or the quality of treatment provided to ethnic communities. The General Medical Council emphasises the importance of “system-wide action” to equip doctors with the skills and knowledge necessary to effectively meet the needs of diverse communities [58]. The studies that received a low or medium rating were primarily criticised for lacking granularity in the research process and analysis. Furthermore, although most studies included quantitative data, they often did not use clear statistical tests to determine significance, where applicable. Three studies received a high rating, as they demonstrated a clear research aim, interpretation of results and thorough analysis during the review [23,27,29].

## 5. Conclusions

Ethnic disparities in burn injuries in the UK highlight notable differences in both incidence and outcomes among various communities. Asian individuals, in particular, experience higher rates of burn injuries, with distinct patterns and types compared with other ethnic groups. Culturally specific factors, such as traditional cooking practices and household environments, poverty and high-density dwellings, could contribute to these variations. These findings highlight the need for developing culturally sensitive prevention strategies. Furthermore, differences in the delivery of medical care, such as the quality of first aid provided and variations in length of hospital stay, suggest that existing protocols may not adequately address the needs of ethnically diverse populations. Addressing these disparities requires a comprehensive approach that includes culturally sensitive education programmes for burn prevention, promotion of appropriate first aid practices, improved access to timely and effective medical care, and targeted interventions to better understand the underlying causes. However, further research is required to enhance our understanding of burn injuries and the quality of care across other regions of the UK not covered in this report. Future research should include long-term treatment follow-up of burn patients, apply robust statistical analyses and ensure consistent classification of ethnicity. By focusing on these areas, it is possible to achieve more equitable health outcomes in the assessment and treatment of a burn injury.

## Figures and Tables

**Table 1:** The table shows the list of included studies and their research report characteristics. * Methodology column: 1 = Type of study, 2 = Type of data analysis. * Sample Size, Age & Gender column: 1 = Total number of individuals, 2 = Mean age (age range) in years, 3 = Males (%), Females (%), Gender not specified (%). Ethnicity column: Numbers reported in each study in descending order (%). * Cause of Burn column: 1 = Cause of Burn, 2 = Mode of Burn (e.g., Accidental, Work-related or Intentional). UV = Ultra-violet Radiation. SIR = Standardised Incidence Ratios; IMD = Index of Mass Deprivation; TBSA = Total Body Surface Area; OR = Odds Ratio; CI = Confidence Interval; N/S = Not Stated. ∴ The database study encompassed data from 1991 to 2010; however, ethnicity-related variables were analysed from 2001 to 2010. ^‡^ These studies used the same dataset but only one was included in any calculation. ° Non-Asian individuals were classed into the unknown section of ethnicity.

Author	Title	Study Aims	* Methodology	Timeline	* Sample Size, Age & Gender	Ethnicity	* Cause of Burn	Outcomes
Studies that discussed Ethnic Disparities in Infants, Children and Young Adults								
Alnababtah et al., 2011 [20]	Burn injuries among children from a region-wide paediatric burns unit	Patterns in age, gender, ethnicity, IMD, season, time, cause and severity of burns (TBSA), alongside length of hospital stay	Retrospective observational study Descriptive statistics and one-tailed Chi-squared tests	2004–2008	1249 4 (0 to 16) 744 (60), 504 (40)	White: 787 (63) Asian: 353 (28) African: 75 (6) Mixed: 34 (3)	Scald = 835, Contact = 202, Flame = 118, Flash = 30, Unknown = 28, Chemical = 12, Electrical = 10, Friction = 7, UV = 4, Other = 3 N/S	The primary source of burns were spills (765 cases; 61%) and contact injuries (150 cases; 12%). Asian British children exhibited higher rates of admission for burns and were significantly younger at the time (*p* < 0.05).
Alnababtah and Khan 2017 [21]	Socio-demographic factors which significantly relate to the prediction of burns severity in children	Relationship between age, gender, ethnicity and the incidence and mechanisms of burn injuries	Cross-sectional study Kruskal Wallis, Chi-squared tests and logistic regression	2011–2012	160 N/S (≤5 to >15)) 82 (51), 78 (49)	White British: 78 (49) Asian British: 40 (25) African British: 15 (9) Other: 12 (8) Afro-Caribbean: 10 (6) Mixed: 5 (3)	Scald = 119, Contact = 30, Other = 11 N/S	Burn injuries were significantly higher in children ≤ 5 years old (*p* < 0.001) and male children (58.1%). Burns were more frequent in minority ethnic groups (*p* < 0.001); younger aged parents ≤ 25 years old (*p* = 0.048); and children living with single parents (*p* = 0.001). Most burns cases resulted from spills (74.4%) and during mealtimes (*p* < 0.001).
Graham et al., 2012 [22]	Are parents in the UK equipped to provide adequate burns first aid?	Differences in first aid practice for burns, including duration of running cool water, seeking medical attention or use of cling film or inappropriate remedies by demographic	Cross-sectional survey Descriptive statistics and Chi-squared tests	2009	188 N/S (<20 to >40) N/S	White: 152 (81) Other: 36 (19)	N/S N/S	White British parents were significantly more likely to administer appropriate first aid using cool water compared to other ethnic groups (*p* = 0.05). 92% (n = 173) of all parents reported using appropriate dressings to protect the wound, 26% (n = 9) of parents from minority ethnic backgrounds indicated the use of potentially harmful remedies.
Richards et al., 2017 [23]	A five-year review of paediatric burns and social deprivation: Is there a link?	Associations between age, gender, ethnicity IMD, mechanism of burn and incidence, alongside first aid practices	Retrospective observational study Descriptive statistics and Pearson’s correlation co-efficient, Fisher’s exact test and Chi squared test	2006–2011	1688 N/S (0 to 15) 985 (58), 703 (42)	White: 760 (45) Asian: 456 (27) African: 169 (10) Unknown: 303 (18)	Scalds = 1038, Contact = 426, Flame = 115, Flash = 27, Chemical = 20, Electrical = 12, Unknown = 50 N/S	The most common mechanism of injury was scalding (61%) and there was a male preponderance (58%). The most affected age group were 1–2-year-olds (38%). Children from Asian and African descent were over-represented in hospital admissions for burn injuries (*p* = 0.0065).
Tan et al., 2012 [24]	Ethnic differences in burn mechanism and severity in a UK paediatric population	Differences in age, gender, ethnicity, IMD cause and location of burn, severity and mechanism of burn and length of hospital stay	Retrospective observational study Chi squared tests, one-way ANOVAs and Spearman rank test	2005–2010	766 4.5 (0 to 16) 463 (60), 303 (40)	White: 692 (90) Asian: 19 (3) Other: 18 (2) Chinese: 16 (2) Black: 13 (2) Mixed: 8 (1)	Scalds = 469, Contact = 151, Flame = 61, Flash = 54, Chemical = 15, Electrical = 7, Friction = 6, UV = 3 N/S	Ethnic minority children sustained burns with a significantly higher total body surface area (*p* < 0.001) and experienced longer hospital stays (*p* < 0.001) compared to non-ethnic minority children and ethnic minority children were found to be more socioeconomically deprived than their non-ethnic minority counterparts (*p* = 0.02).
Vipulendran et al., 1989 [25]	Ethnic differences in incidence of severe burns and scalds to children in Birmingham	The incidence of burns by age, gender, ethnicity, TBSA	Retrospective observational study Descriptive statistics	1983–1987	600 N/S 365 (61), 235 (39)	Non-Asian °: 445 (74) Asian: 155 (26)	Scalds = 446, Other = 154 N/S	A disproportionate number of Asian children were admitted for burn injuries in Birmingham compared to their non-Asian counterparts (OR: 1.55; 95% CI: 1.29–1.8)
Studies that discussed Ethnic Disparities in Adults (and Infants to Children)								
Brewster et al., 2013 [26]	Trends in hospital admissions for burns in England, 1991–2010	Associations between sex, age, ethnicity, IMD quintile and burns	Descriptive population-based study Gender-specific and age-standardised burn admission rates stratified by ethnicity	2001–2010 ∴	188,597 N/S (0 to 85) N/S	N/S	N/S N/S	Rates of hospital admissions for burn injuries in England were higher in most ethnic minority groups, compared to White British population.
Heng et al., 2015 [27]	Geographical analysis of socioeconomic factors in risk of domestic burn injury in London 2007–2013	Risk of burn with age, gender, ethnicity, IMD, health deprivation and disability score, household density and barriers to housing	Retrospective ecological analysis SIRs were calculated for each area, with Bayesian modelling and multivariate regression used to analyse relative risks	2007–2013	2100 N/S (0 to 14 and 15+) 1157 (55), 929 (44), 14 (0.7)	N/S	N/S N/S	The relative risk of paediatric domestic burn injury was independently associated with percentage of ethnic minorities (*p* = 0.005), income deprivation (*p* < 0.001), health deprivation and disability (*p* = 0.031) and percentage of families with ≥3 children (*p* = 0.004).
Rawlins et al., 2006 ^‡^ [28]	Burn patterns of Asian ethnic minorities living in West Yorkshire, UK	Patterns in age, gender, ethnicity, occupation, cause and type of burn, burn location, first-aid, extent of burn, pre-hospital analgesia and outcomes	Prospective observational study Descriptive statistics	2003–2004	460 23 (0 to >61) 271 (59), 189 (41)	White: 263 (57) Asian: 188 (41) Black: 9 (2)	Scald = 249, Contact = 106, Flame/Fire = 74, Chemical = 28, UV = 3 Accidental = 349, Work-related = 106, Intentional = 5	In the Asian ethnic minority group, 37% of contact burns were caused by hot irons, and 11% of patients used inappropriate remedies such as butter and toothpaste. There were no significant differences in burn severity or mortality compared to non-Asian patients.
Shepherd et al., 2023 [29]	Associations between Ethnicity and Referrals, Access and Engagement in a UK Adult Burns Clinical Psychology Service	Access & engagement with burns clinical psychology in respect to age, gender, ethnicity	Retrospective cohort Pearson’s Chi-square, Fisher’s exact, and Kruskal–Wallis tests	2014–2022	699 43 (16 to 97) 383 (55), 315 (45)	White: 471 (67) Asian: 43 (6) Other White: 38 (5) Black: 28 (4) Mixed: 10 (1) Other: 9 (1) Unknown: 100 (14)	N/S N/S	White British patients (*p* < 0.001) were less likely to be referred to the burns clinical psychology service, whereas patients within Black (*p* < 0.001) and Asian (*p* < 0.001) ethnic groups were more likely to be referred.
Khan et al., 2007 ^‡^ [30]	The Bradford Burn Study: the epidemiology of burns presenting to an inner-city emergency department	Relationship between age, gender, ethnicity, occupation, cause and type of burn, alongside burn location, monthly variations, severity, and outcomes, with the use of pre-hospital analgesia in burn patients	Prospective observational study Descriptive statistics	2003–2004	460 23 (0 to >61) 271 (59), 189 (41)	White: 263 (57) Asian: 188 (41) Black: 9 (2)	Scald = 249, Contact = 106, Flame/Fire = 74, Chemical = 28, UV = 3 Accidental = 349, Work-related = 106, Intentional = 5	Although individuals of Asian origin represent only 10.0% of the population, they accounted for 40.8% of burn injuries, in contrast to 57.1% among White patients. Most cases (85%) were accidental, with scalds representing the most common injury type at 52%.

## Data Availability

No new data were created or analysed in this study. Data sharing is not applicable to this article and the study was not registered.

## Supplementary Materials

The following supporting information can be downloaded at: https://www.mdpi.com/article/10.3390/ebj6030048/s1, Table S1: The quality appraisal summary of the included studies; Table S2: Line-by-line advance search history.

## Abbreviations

The following abbreviations are used in this manuscript:

TBSA	Total Body Surface Area
NHS	National Health Service
PRISMA	Preferred Reporting Items for Systematic Reviews and Meta-Analysis
CASP	Critical Appraisal Skills Programme
IMD	Index of Mass Deprivation
SIR	Standardised Incidence Ratios
ONS	Office for National Statistics
