# Supplementary material for: Exploring Ethnic Disparities in Burn Injury Outcomes in the UK: A Systematic Review
Source: Eur Burn J. 2025 Aug 22;6(3):48. doi: 10.3390/ebj6030048 (PMC12452468; doi:10.3390/ebj6030048)
Supplement: Supplementary file 1 [file ebj-06-00048-s001.zip › ebj-3752619-supplementary.pdf]

Supplementary Data

Table S1. The quality appraisal summary of the included studies.

	Brewster (2013)	Heng (2015)	Khan et al (2007)	Rawlins (2006)	Shepherd (2023)	Graham (2012)	Alnababtah (2017)	Alnababtah (2011)	Richards (2017)	Tan (2012)	Vipulendran (1989)
Did the study address a clearly focused issue?	Yes	Yes	Yes	Yes	Yes	Yes	Yes	Yes	Yes	Yes	Yes
Did the authors use an appropriate method to answer their question?	Yes	Yes	Yes	Yes	Yes	Yes	Yes	Yes	Yes	Yes	Yes
Were the cases or cohort recruited in an acceptable way?	Yes	Yes	Yes	Yes	Yes	Can't tell	Yes	Yes	Yes	Yes	Can’t tell
Was the exposure accurately measured to minimise bias?	Yes	Yes	No	No	Yes	Yes	Can't tell	Can't tell	Yes	Yes	No

Have the authors taken account of the potential confounding factors in the design and/or in their analysis?	Can’t tell	Yes	Can’t tell	No	Yes	No	Yes	No	No	No	No
Are the results of the study clear?	Yes	Yes	Yes	Yes	Yes	Yes	Yes	Yes	Yes	Yes	Yes
Were the results precise enough?	No	Can't tell	Can't tell	Can't tell	Yes	Can't tell	Can't tell	Can't tell	Yes	Yes	No
Can the results be applied to the local population?	Yes	Yes	Can't tell	Can’t tell	Can't tell	No	No	Yes	Yes	No	No
Do the results of this study fit with other available evidence?	Yes	Yes	Yes	Yes	Can't tell	Yes	Yes	Yes	Yes	Yes	Yes
No conflict of interest? (‘Yes’ indicates that no conflict of interest was reported; ‘No’ indicates that a conflict of interest was disclosed)	Yes	Yes	Yes	Can't tell	Yes	Can't tell	Can't tell	Yes	Yes	Yes	Can’t tell
core (0 to 20)	17/20	19/20	16/20	13/20	18/20	13/20	15/20	16/20	18/20	16/20	10/20

Table S2. Line-by-line advance search history

	PubMed	TRIP	Ovid	EBSCO
Search Terms	("Ethnic" or "Race" or "Black" or "Asian" or "Minority" or "Mixed" or "Other" or "BAME") .af.<br><br>AND<br><br>("Burn" or "Burn patient" or "Burn outcome" or "Burn access" or "Burn barriers" or "Burn treatment" or "Burn management") .af.<br><br>AND<br><br>("United Kingdom" or "Britain" or "England" or "Wales" or "Scotland" or "Northern Ireland") .af.	("Ethnic" or "Race" or "Black" or "Asian" or "Minority" or "Mixed" or "Other" or "BAME") .ds.<br><br>AND<br><br>("Burn" or "Burn patient" or "Burn outcome" or "Burn access" or "Burn barriers" or "Burn treatment" or "Burn management") .ds.<br><br>AND<br><br>("United Kingdom" or "Britain" or "England" or "Wales" or "Scotland" or "Northern Ireland") .ds.	1. ("Ethnic" or "Race" or "Black" or "Asian" or "Minority" or "Mixed" or "BAME") .ab.<br><br>2. ("Burn" or "Burn patient" or "Burn outcome" or "Burn access" or "Burn barriers" or "Burn treatment" or "Burn management") .af.<br><br>3. ("United Kingdom" or "Britain" or "England" or "Wales" or "Scotland" or "Northern Ireland")<br><br>4. 1 and 2 and 3<br><br>5. Limit 4 to Full Text	1. ("Ethnic" or "Race" or "Black" or "Asian" or "Minority" or "Mixed" or "Other" or "BAME") .af.<br><br>2. ("Burn" or "Burn patient" or "Burn outcome" or "Burn access" or "Burn barriers" or "Burn treatment" or "Burn management") .af.<br><br>3. ("United Kingdom" or "Britain" or "England" or "Wales" or "Scotland" or "Northern Ireland")<br><br>4. 1 and 2 and 3<br><br>5. Limit 4 to Full Text
Filters Applied	Full Text<br><br>English	Full Text<br><br>English	All Database & Resources<br><br>English	All Databases<br><br>English

*Variations to search terms were applied to ensure a wide range of results. Af = All fields; Ab = Abstract; Ds = Document search; BAME = Black, Asian, Minority ethnic. 'AND' and 'OR' Boolean operators were applied to combine searches, refining the database further. The same terms were used for each database for consistency. Where applicable, the "English" language was selected.
